# Supplementary material for: Comparing two protocols of shock wave therapy for patients with plantar fasciitis: A pilot study
Source: PLoS One. 2024 May 6;19(5):e0302553. doi: 10.1371/journal.pone.0302553 (PMC11073689; doi:10.1371/journal.pone.0302553)
Supplement: S1 File — (PDF) [file pone.0302553.s002.pdf]

A randomized controlled trial comparing two protocols of shock wave therapy for patients with planter fasciitis.

By:

Fatima Ali Alkalbani

U20105893

CHAPTER I
INTRODUCTION

Background:

Between 1995-2000, the number of patient visits to the outpatient departments was highly increasing, reaching around one million visits annually for the diagnosis and management of plantar fasciitis (PF). However, 19% of all visits were made for physiotherapy, 26% for exercises counseling and the other percentage was divided between pain medication and orthopedic surgeons in the National Hospital Ambulatory Medical Care **(Riddle et al., 2004).**

The heel pain at the inferior and medial aspect of the calcaneus effects millions of people in the world. It is estimated that the prevalence of tenderness on palpation with foot pain related to the planter fascia 6.4 for men and 7.2% for women. Similarly, the plantar heel pad was 4.3% for men and 3.9% for women. **(J. E. Dunn et al.,2004).**

Furthermore, PF is considered as a common cause of heel pain which effects on the patient's life. However, the plantar fasciitis impacts both genders but the percent of the incidence with women is slightly more than men as reported earlier. It is associated with sports but commonly reported with runners and approximately the percent of incidence is 5% - 10%. The high percent of incidence presents with runners and is considered normal due to the running's biomechanics. During running the vertical force of the foot against the ground can lead to double the body weight acting on the PF and foot arch which should be responsible for the absorption mechanism. However, the cost management of plantar fasciitis was estimated between 192-376 million in the United States **(Petraglia et al.,2017).**

Most of the studies analyzed different treatments of plantar fasciitis including the shockwave but with no specific or fixed parameters set for the administration of the shockwave. It was previously demonstrated that shockwave therapy have a significant improvement in the pain that confirmed by using analog visual scale outcome which seen after three weeks without specified the parameters of shockwave **(Leao et al, 2020).**

Another study compared the effect of shockwave therapy and local corticosteroid injection on patients of plantar fasciitis that confirmed both of them contributes in decreasing the pain of plantar fasciitis but shockwave therapy given longer relief than the local corticosteroid injection. However, there is no identified a fixed parameters of shockwave in this study (**Xu et al, 2020).**

Statement of the problem:

What is the difference between two protocols of shockwave therapy on planter fasciitis with regards improving function and decreasing the pain?

Purpose of the study:

The purpose of this study is to compare the results of different protocol of extracorporeal shockwave therapy on improving function with plantar fasciitis and reducing the pain after 6 sessions of the intervention.

Delimitation:

The study will be delimitated to:

1. Patients with plantar fasciitis of both genders
2. The age will be ranged from 20 to 50 years old.
3. The patients should be able to understand and follow the verbal instructions.
4. The pain should be from moderate to severe.
5. Functional limitation from moderate to severe

Basic assumptions:

1. All subjects will attend the assessment sessions
2. All the subjects will receive the intervention as established
3. The modality will be properly calibrated

Significance of the study:

To provide evidence that both protocols are effectiveness but there is lacking in the previous literature about the difference between 2 protocols when using the shock wave when handling cases of PF.

Shockwave therapy is a safe and effective non-surgical treatment of plantar fasciitis comparing to other treatments. Moreover, the researchers recommend the use of shockwave therapy for plantar fasciitis.

This study may help physiotherapists to exclude using the slow acting devices and save the time by using a specific protocol of a fast effective modality in the treatment of PF.

Hypothesis:

There will be a significant difference between different protocols of shockwave on function and pain in cases of PF.

CHAPTER II
LITERATURE REVIEW

The literature review of this study will be presented as follows:

1. Definition of plantar fasciitis.
2. The risk factors of plantar fasciitis.
3. Anatomy of plantar fascia.
4. The pathophysiology of plantar fasciitis.
5. Shockwave therapy

Plantar fasciitis:

The plantar fascia is a thick band that connects the calcaneus bone to the forefoot. The plantar fascia absorbs the shock for pressure on the foot and helps in the walking by supporting the arch of foot. Consequently, the plantar fascia can be damaged because of the pressure during the daily life. Plantar fasciitis is a degenerative condition which is results from reparative micro tears in the fascia which lead to an inflammation in the insertion of the plantar fascia. The plantar fasciitis is the one of causes of heel pain and orthopedic complaints. The

pain actually in the bottom of the heel and sometime in the medial aspect of the heel **(Luffy et al.,2018).**

Risk factors :

The risk factors of plantar fasciitis are divided to intrinsic and extrinsic. The intrinsic factors consist of anatomic factors such as (shortness of Achilles tendon, obesity, pes planus and pes cavus) , Also the biomechanics' factors considered part from intrinsic factors such as (restricted dorsiflexion of ankle, excessive foot pronation, weakness of intrinsic foot and plantar flexors muscles). So, the extrinsic factors consist of environmental factors such as (inappropriate shoes, walking on hard surface, barefoot , prolong standing/walking and poor alignment). All these factors can assessed by physiotherapist in the examination session that guides to appropriate treatment **(Schwartz et al.,2014).**

Anatomy:

The plantar fascia **(Fig.1)** includes three bands of thickened fibrous aponeurosis, which are dividing depend on their location. The divided bands are the medial, central and lateral which is the central considers the thickest one and it stronger more than others. However, the plantar fascia originate at calcaneus tubercle from medial aspect and runs forward as fan shape to insert at each proximal phalanx which it dividing into five bands and continuing to forming the flexor sheaths of the toes. The plantar nerves are moves in and around of plantar fascia. The plantar fascia helps in stability of first metatarsophalangeal joint and arch during the Windlass Mechanism and helps in normal walking as well mainly thru heel rise **(Thompson JV et al.,2014).**

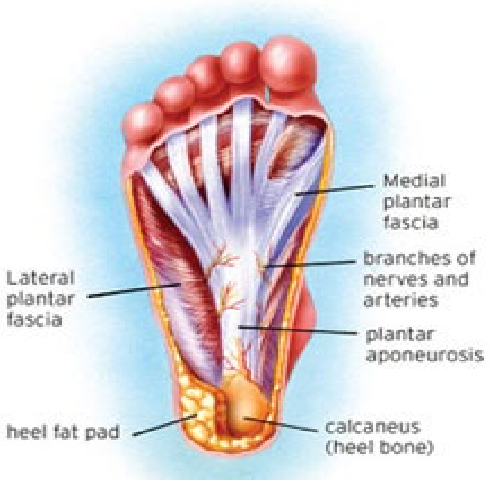

Anatomy of the Sole of the Foot

Fig.1: Anatomy of the sole/plantar fascia

Pathophysiology:

Plantar fasciitis is a degenerative process including the thickened fibrous aponeurosis (plantar fascia) and most common site of degenerating is a medial side of calcaneus tubercle. The process is happen through the repetitive strain in the tissue which lead to micro-tearing of plantar aponeurosis. Furthermore, the histologic analysis confirmed that a calcification, chondroid metaplasia and collagen necrosis results the fibrosis and thickening of plantar fascia. Plantar fasciitis assumed to be an inflammation process, but the findings (thickening and fibrosis of plantar) suggested a degenerative mechanism. Depending on that, the authors suggested a plantar fasciosis could be more accurate term rather than plantar fasciitis **(Lemont H et al.,2003).**

Extracorporeal Shockwave Therapy (ESWT):

Shockwave therapy considered a multidisciplinary modality that is use in different clinics such as (physiotherapy, orthopedics and sport medicine). So, Shockwave therapy is an non-invasive treatment of damaged soft tissue. In addition, it has other features like a safe, fast and effective treatment to relief the pain. However, the clinicians preferring to use it before the decision for surgery, injections and steroid medication to manage the pain and mobility restoration that is clinically confirmed to stimulate metabolic reactions. The shockwave therapy stimulate the enzymes and provoke an acute healing process which means activates the process of healing and decreasing the pain. All the application of shockwave therapy is an evidence-based and it is an effective treatment for many conditions such as plantar fasciitis, tennis elbow and Achilles, hamstring and gluteal tendons. Hence, the shockwave therapy works after determine the complaint area by the clinician after that should put some gel on the area then the shockwave device is apply. The shockwave device consist of hand piece and fast moving rocket which controlled by a compressor. The rocket transmits the force to the head that produce the shockwaves then move in and absorbs by the body. Addition to that the clinicians can adjust the frequency, impulse, pressure and time of wave **(Cheing et al.,2003).**

There are many studies talking about shockwave therapy in the treatment of PF. The researchers showed a significant clinical improvement of focused extracorporeal shockwave therapy in the treatment of PF without using anesthesia and confirmed between 50 – 65% of success rate (**Gollwitzer H et al, 2015).**

There is another study recommended to use shock wave therapy in the treatment of PF to any patient over 6 months. Moreover, it confirmed the significant improvement and no negative side effects have been reported from extracorporeal shock wave therapy. So, the results of this study revealed that shock wave therapy is useful, safe and effective with no negative side effects (**Rompe et al, 2003**).

CHAPTER III
METHODOLOGY

Subjects:

Patients with PF will participate in this study and will be selected based on the following criteria:

Inclusion criteria:

The patient will be included in this study if they meet of the following criteria

1. The participants should present with unilateral pain
2. The participants' age will be ranged from 20-50 years old.
3. The participants should have the pain from for at least 6 weeks.
4. Moderate disability as assessed by the foot function index (FFI)
5. The Body Mass Index (BMI) should be normal.
6. The participants should have pronated feet (6-9 on the foot posture index).

Exclusion criteria:

<u>The patients will be excluded from this study if they meet any of the following criteria</u>

1. History of surgery or fracture
2. History of corticosteroid injection within 6 months
3. Unable to follow or understand the instructions
4. High BMI
5. Sever foot pronation (+10 on the foot posture index)

Trial design:

A pre-test post-test randomized control trail.

Ethical consideration:

This research will be approved by the Research Ethical Committee (REC)/UoS. Consent forms will be distributed to participants prior to data collection. The participants will be informed that their data will be used for the study only. Privacy and confidentiality will be maintained by using coding system for data. The data will be stored on a locked-password-required computer in the primary investigator's office and will be accessed by the study's researchers only. Participation in this study is completely voluntary. The participant has the right to leave the study at any time. This will not result in any penalty or loss of benefits to which they are entitled to. If the participant decides to withdraw, any data taken from them will not be used in the research and will be destroyed.

Sample size estimation:

A pilot study will be conducted to detect the effect size required (at alpha level of 0.05 and power of 0.8)

A priori power analysis will be performed (G*power, version 3.1.9.3, Duesseldorf, Germany), based on previous studies on PF

Pain, considered the Primary outcome measure and revealing a visual analogue scale (VAS) of 4-10 on the pain scale for PF.

Function limitation considered the secondary outcome measure and exposing a Foot Function Index of 100/170 or at least 60%.

Randomization (Recruitment and allocation):

Recruitment

The patients with plantar fasciitis will be recruited from the Physical Therapy and Rehabilitation Center (out-patient) in Alain hospital, UAE. The purpose of the study will be explained to all participants before the start the assessment. A written consent forms will be signed after explanation.

Ethical approval will be obtained from education department of Alain hospital or from the University of Sharjah

Randomization & Allocation

In this study, patients will be randomly divided into two groups of equal number.

Restricted randomized sampling will be used in this study for randomization of the participants into three groups. Using permuted block randomization, with 1:1:1 allocation ratio. In a three group design, Blocks having equal numbers of As, Bs and Cs (A = intervention 1 and B = intervention 2, and C= control) are used, with the order of treatments within the block being randomly permuted

The groups will be:

<u>Intervention group A:</u>

The patients in this group will receive selected physical therapy program combined with shockwave therapy (ESWT) of these parameters :

1. Frequency : (15 Hz) on heel
2. Intensity / pressure: 3
3. Impulses 1800
4. Number of Sessions : 6 session (1 per week)

<u>Intervention group B:</u>

The patients in this group will receive the same selected physical therapy program combined with shockwave therapy (ESWT) but with different parameters :

1. Frequency : (10 Hz) on heel
2. Intensity / pressure: 4
3. Impulses 1800
4. Number of Sessions : 6 session (1 per week)

Control group - C:

This group will receive the same physiotherapy program in addition to sham shock wave therapy

The pain and function improvement will be assessed before and after 6 sessions. An additional assessment will be performed at the end of the 12th week for a short follow-up

Blinding:

In this study:

- The participants will be blinded from the parameters of the intervention used.

Data collection:

The mean and Standard Deviation of pain scores which will be calculated before, during and after the intervention for all participants in both group.

- First assessment will be done before the intervention
- Second assessment will be done after 3 sessions
- Follow up assessment after the complete 6 sessions

Statistical method:

For the statistical analysis of the information gathered SPSS Statistics (IBM Corp. Released 2020. IBM SPSS Statistics for Windows, Version 27.0. Armonk, NY: IBM Corp) will be used. The frequency, mean, and standard deviation or median and interquartile range will be calculated for the tested

variables. The data will be collected from the participants before and after starting the program.

The paired t-test will be used for within group comparisons while the unpaired will be used for the between group comparisons.

Interventions:

Regular physiotherapy program to be introduced to both groups:

<u>The stretching exercise program:</u>

The program will consist of plantar fascia, gastrocnemius and soleus muscles. The stretching exercise for all these tissues will be in standing position front the wall then let the patient to move the painful feet back of the normal feet, and front knee slightly flexed. The therapist will ask the patient to keep the trunk and knee straight and stabilize the heel on the floor then lean toward the wall till feel the stretch all along the calf of the painful feet and hold the stretch for 20 to 30 seconds. This stretching exercises will done 5 times for 5 set per a day for 6 weeks (**Garrett TR et al, 2013**).

<u>The strengthening exercise program:</u>

The program will consist of a toe curl exercises, Marble pickup exercises, small ball roll exercises and heel raise exercises. The toe curl, marble pickup and small ball roll exercises will be instructed to the patients to sit comfortably on the chair with both feet on the floor, hence with toe curl exercises the therapist will put a small towel or tissue on the floor front the painful feet then the patients scrunch the toes to pull the towel or tissue toward them. The marble pickup exercises, the therapist will place 10 marbles and small box front the patient's feet and ask the patient to pick up one marble by using the toes and place it in the small box. A small ball roll exercises, the therapist will place small ball (tennis ball) front the patient's feet and ask the patient to roll it around for 2 minutes. The heel raise exercises will be instructed to the patient by ask the patient to stand front the wall with both feet on the floor then raise both heels so the patient will standing on the toes, if need support to do the exercises let the patient to stead on the wall with two hands. All these exercises will apply it 5 times in 5 set per a day for 6 weeks. This study showed that the effectiveness of strengthening exercises in reducing the pain with plantar fasciitis patients (**Thong-On et al, 2019**).

Extracorporeal shockwave therapy (ESWT):

Extracorporeal shockwave therapy (physiotur) with Handpiece 12 mm will be set for the study group with the continues mode, frequency 15 Hz on heel, pressure of 3 and impulses of 1600. On the other hand, the parameter for controlled group will be set with frequency 10 Hz on heel, pressure of 4 and impulses of 800. Then apply it to the patients on painful area of the heel at different points (center and medial aspect of the heel) and will apply 200 impulses in each painful points till complete 1600 impulses with patients in the study group and 800 impulses with patients in the control group. The patients will be on prone position with rest both legs on the sigmoid wedge.

The program of strengthening and stretching exercises will be same for both groups combined with shockwave therapy but with different parameter in each group. Both groups will receive the intervention six times (one time per week over two months) and will follow with home exercises program.

Outcomes:

- **Foot Function Index:**

The foot function index (FFI) is an outcome measure that has been used to measure the impact of foot pathology and it can be used in clinical and studies setting which related to foot disorders. FFI is a questionnaire consist of 23 self-

reported items and divided to sub-scales. The patient has to score him self in each question from 0 (no pain/disability) to 10 (worst pain/required help). The pain sub-scale consist of 9 questions that it measures the pain in different situation. The second sub-scale is a disability which consist of 9 questions and it measures the difficulty performing. The last sub-scale is activity limitation which consist of 5 questions and it measures the limitation or restriction of activities (**Budiman-Mak E et al, 1991)**

Additionally, the FFI is recommended as a validated and reliable outcome scale for using in foot pathologies intervention trails (**Saag KG et al,1996)**.

- **Visual Analog Scale**

The pain will be assessed before and after the intervention by using visual analog scale (VAS) to measure the effectiveness of the treatment. The visual analog scale is a validated outcome measure has been used to measure the pain and it widely using in the researches. VAS is a measurement instrument for acute and chronic pain. It is a 10 cm line which represents range between two points (the left point is 0 cm that means no pain and the right point is 10 cm which means is the worst pain) and the score will be recorded by making a mark on the line and it interpreted as the patient's pain **(Delgado et al.,2018).**

Addition to that, the reliability of the VAS seems to be very high. Whereas, there are a studies confirmed that the VAS is adequately reliable for acute measurement **(Bijur PE et al.,2001)**.

References :

Riddle, D. L., & Schappert, S. M. (2004). Volume of Ambulatory Care Visits and Patterns of Care for Patients Diagnosed with Plantar Fasciitis: A National Study of Medical Doctors. *Foot & Ankle International*, *25*(5), 303–310. https://doi.org/10.1177/107110070402500505

J. E. Dunn, C. L. Link, D. T. Felson, M. G. Crincoli, J. J. Keysor, J. B. McKinlay, Prevalence of Foot and Ankle Conditions in a Multiethnic Community Sample of Older Adults, *American Journal of Epidemiology*, Volume 159, Issue 5, 1 March 2004, Pages 491–498, https://doi.org/10.1093/aje/kwh071

Petraglia, F., Ramazzina, I., & Costantino, C. (2017). Plantar fasciitis in athletes: diagnostic and treatment strategies. A systematic review. *Muscles, ligaments and tendons journal*, *7*(1), 107–118. https://doi.org/10.11138/mltj/2017.7.1.107

Leao, R. G., Azuma, M. M., Ambrosio, G., Faloppa, F., Takimoto, E. S., & Tamaoki, M. (2020). Effectiveness of shockwave therapy in the treatment of plantar fasciitis. *Acta ortopedica brasileira*, *28*(1), 7–11. https://doi.org/10.1590/1413-785220202801227402

Xu, D., Jiang, W., Huang, D., Hu, X., Wang, Y., Li, H., Zhou, S., Gan, K., & Ma, W. (2020). Comparison Between Extracorporeal Shock Wave Therapy and Local Corticosteroid Injection for Plantar Fasciitis. Foot & Ankle International, 41(2), 200–205. https://doi.org/10.1177/1071100719891111

Luffy, Lindsey MSPAS, PA-C; Grosel, John MD; Thomas, Randall DPM; So, Eric DPM Plantar fasciitis, Journal of the American Academy of Physician Assistants: January 2018 - Volume 31 - Issue 1 - p 20-24 doi: 10.1097/01.JAA.0000527695.76041.99

Schwartz, E. N., & Su, J. (2014). Plantar fasciitis: a concise review. *The Permanente journal*, *18*(1), e105–e107. https://doi.org/10.7812/TPP/13-113

Thompson JV, Saini SS, Reb CW, Daniel JN. Diagnosis and management of plantar fasciitis. J Am Osteopath Assoc. 2014 Dec;114(12):900-6. doi: 10.7556/jaoa.2014.177. PMID: 25429080.

Lemont H, Ammirati KM, Usen N. Plantar fasciitis: a degenerative process (fasciosis) without inflammation. J Am Podiatr Med Assoc. 2003 May-Jun;93(3):234-7. doi: 10.7547/87507315-93-3-234. PMID: 12756315.

Gollwitzer H, Saxena A, DiDomenico LA, Galli L, Bouché RT, Caminear DS, Fullem B, Vester JC, Horn C, Banke IJ, Burgkart R, Gerdesmeyer L. Clinically relevant effectiveness of focused extracorporeal shock wave therapy in the treatment of chronic plantar fasciitis: a randomized, controlled multicenter study. J Bone Joint Surg Am. 2015 May 6;97(9):701-8. doi: 10.2106/JBJS.M.01331. PMID: 25948515.

Rompe, Jan & Decking, Jens & Schoellner, Carsten & Nafe, Bernhard. (2003). Shock Wave Application for Chronic Plantar Fasciitis in Running Athletes A Prospective, Randomized, Placebo-Controlled Trial. The American journal of sports medicine. 31. 268-75. 10.1177/03635465030310021901.

Garrett TR, Neibert PJ. The effectiveness of a gastrocnemius-soleus stretching program as a therapeutic treatment of plantar fasciitis. J Sport Rehabil. 2013 Nov;22(4):308-12. doi: 10.1123/jsr.22.4.308. Epub 2013 May 22. PMID: 23752554.

Thong-On, S., Bovonsunthonchai, S., Vachalathiti, R., Intiravoranont, W., Suwannarat, S., & Smith, R. (2019). Effects of Strengthening and Stretching Exercises on the Temporospatial Gait Parameters in Patients With Plantar Fasciitis: A Randomized Controlled Trial. *Annals of rehabilitation medicine*, *43*(6), 662–676. https://doi.org/10.5535/arm.2019.43.6.662

Cheing, Gladys & Chang, Hua. (2003). Extracorporeal Shock Wave Therapy. The Journal of orthopaedic and sports physical therapy. 33. 10.2519/jospt.2003.33.6.337

Budiman-Mak E, Conrad KJ, Roach KE. The Foot Function Index: a measure of foot pain and disability. J Clin Epidemiol. 1991;44(6):561-70. doi: 10.1016/0895-4356(91)90220-4. PMID: 2037861.

Saag KG, Saltzman CL, Brown CK, Budiman-Mak E. The Foot Function Index for measuring rheumatoid arthritis pain: evaluating side-to-side reliability. Foot Ankle Int. 1996 Aug;17(8):506-10. doi: 10.1177/107110079601700814. PMID: 8863033.

Delgado, D. A., Lambert, B. S., Boutris, N., McCulloch, P. C., Robbins, A. B., Moreno, M. R., & Harris, J. D. (2018). Validation of Digital Visual Analog Scale Pain Scoring With a Traditional Paper-based Visual Analog Scale in Adults. *Journal of the American Academy of Orthopaedic Surgeons. Global research & reviews*, *2*(3), e088. https://doi.org/10.5435/JAAOSGlobal-D-17-00088

Bijur PE, Silver W, Gallagher EJ. Reliability of the visual analog scale for measurement of acute pain. Acad Emerg Med. 2001 Dec;8(12):1153-7. doi: 10.1111/j.1553-2712.2001.tb01132.x. PMID: 11733293.
