# Supplementary material for: Comparing two protocols of shock wave therapy for patients with plantar fasciitis: A pilot study
Source: PLoS One. 2024 May 6;19(5):e0302553. doi: 10.1371/journal.pone.0302553 (PMC11073689; doi:10.1371/journal.pone.0302553)
Supplement: Attachment — Submitted filename: response to reviewers.docx [file pone.0302553.s003.docx]

Dear Reviewer,

Thank you for your thorough revision and helpful comments…

Kindly find below, the authors’ reply on a point-to-point basis.

Kindly

| Comment | Authors’ reply |
| --- | --- |
| L61-62: Does it cause only heel pain? | Added lines 62-64: ‘, with symptoms of stabbing, non-radiating pain in the early morning of the proximal medio-plantar surface of the foot; the pain becomes worse at the end of the day [2].” |
| Materials and methods: It has not been specified if you followed a guideline and, in this case, which one | As this is a pilot study, we followed the “CONSORT 2010 checklist of information to include when reporting a pilot or feasibility trial” which is already one of the journal’s requirements for submission and was uploaded with the supporting files.  In addition, the sample calculation was done in accordance to the study “Hertzog MA. Considerations in determining sample size for pilot studies. Res Nurs Health. 2008;31: 180–191” reference 9 stated in line 115.  All assessment and treatment parameters have references included in text |
| L306: A short description of the anatomical structures and the pathological alterations are needed before talking about the impact of the shock waves on cells. | Added lines 308-312(version without track changes) / 313-317( version with track changes): “This is due to the fact that plantar fasciitis is a biomechanical overuse syndrome resulting in degenerative changes at its attachment to the calcaneus. Histologic examination of samples taken from plantar fascia release surgeries revealed myxoid degeneration with fragmentation and degeneration of the plantar fascia and bone marrow vascular ectasia [2].” |
