# Supplementary material for: Comparing two protocols of shock wave therapy for patients with plantar fasciitis: A pilot study
Source: PLoS One. 2024 May 6;19(5):e0302553. doi: 10.1371/journal.pone.0302553 (PMC11073689; doi:10.1371/journal.pone.0302553)
Supplement: Attachment — Submitted filename: Responses.docx [file pone.0302553.s004.docx]

Dear Reviewer,

Thank you for your thorough revision and helpful comments…

Kindly find below, the authors’ reply on a point-to-point basis.

Kindly note that the line numbers mentioned below are in the copy with track changes

Warm regards,

| Comment | Authors’ reply |
| --- | --- |
| What is the block size used in the permuted block randomization? | Thank you for your comment.  Lines 125 -126: added “permuted block randomization of different sizes (3,6)” |
| The flow chart shows the final number of patients analyzed was 9, 9 and 8 for three groups. But were all 10 patients' baseline measurements analyzed and shown in Table 1? It is not clear how compliance was defined. | Thank you for your comment. The percentages were checked, and correct percentages were edited in text lines: 230-232 as well as in Table 1  Compliance/ adherence was calculated by dividing the number of participants completing the study by the total number of each group . lines 226-227 |
| Because of the small sample size in each group, median+/-IQR should also be reported. Because of the same reason, Shapiro-Wilks test is not appropriate for testing the normality assumption. | Thank you for your comment.  Median and IQR values added to Table 1  With regards to the Shapiro-wilk test, the choice was based on previous literature revealing the Shapiro-wilk appropriate for sample sizes less than 50.    Elliott AC, Woodward WA. Statistical analysis quick reference guidebook with SPSS examples. 1st ed. London: Sage Publications; 2007  Mishra P, Pandey CM, Singh U, Gupta A, Sahu C, Keshri A. Descriptive statistics and normality tests for statistical data. Ann Card Anaesth. 2019 Jan-Mar;22(1):67-72. doi: 10.4103/aca.ACA_157_18. PMID: 30648682; PMCID: PMC6350423.  Ghasemi A, Zahediasl S. Normality tests for statistical analysis: a guide for non-statisticians. Int J Endocrinol Metab. 2012 Spring;10(2):486-9. doi: 10.5812/ijem.3505. Epub 2012 Apr 20. PMID: 23843808; PMCID: PMC3693611. |
| Table 3 reports the comparison between group A and group B as well as the first row in Table 4. First, such information is duplicated so Table 3 could be removed. Second, It is confusing that p-values from the same comparisons in these two tables were not the same. | The table has been removed and replaced with the pairwise comparisons of the KW test |
| Line 251 and Line 269 have duplicated information presented | Duplication removed |
| There are longitudinal measurements observed from each study participants. It is better to draw individual profile of each outcome over time to show the difference visually. More importantly, the statistical analysis such as linear mixed effect model is better to take advantage of such longitudinal design for estimating the difference between groups, which would be more powerful. Such model can also use all available observed data even if some study participants only had baseline measurements recorded | Thank you for this comment…  The linear mixed effect model depends on the assumption for normality which is not available in our case since the data was not normally distributed. |
| There should be more details about future sample size calculation. For example, which group difference was the effect size of 0.25 from, group A vs group C, group B vs group C, or from all three groups? How was the effect size calculated? Which outcome was used in that effect size calculation, FFI or VAS? Which statistical test was used in this sample size calculation? What is the alpha and beta level used in such calculation? | Thank you for your valuable comment…  Added lines 288-292: For future sample size calculations, considering the VAS as the primary outcome, the effect size for post-hoc Kruskal-Wallis test was calculated using the formula r = z/√N (r: effect size; z: z value; N: Observation number).  The effect sizes between the groups were reported as 0.282, 1.09 and 0.796 respectively.  A total sample size of 123 participants was calculated using the G-power software for the ANOVA repeated measures within-between interaction test, assuming an effect size of 0.282, α level of 0.05, and power of 0.95. |
|  |  |
